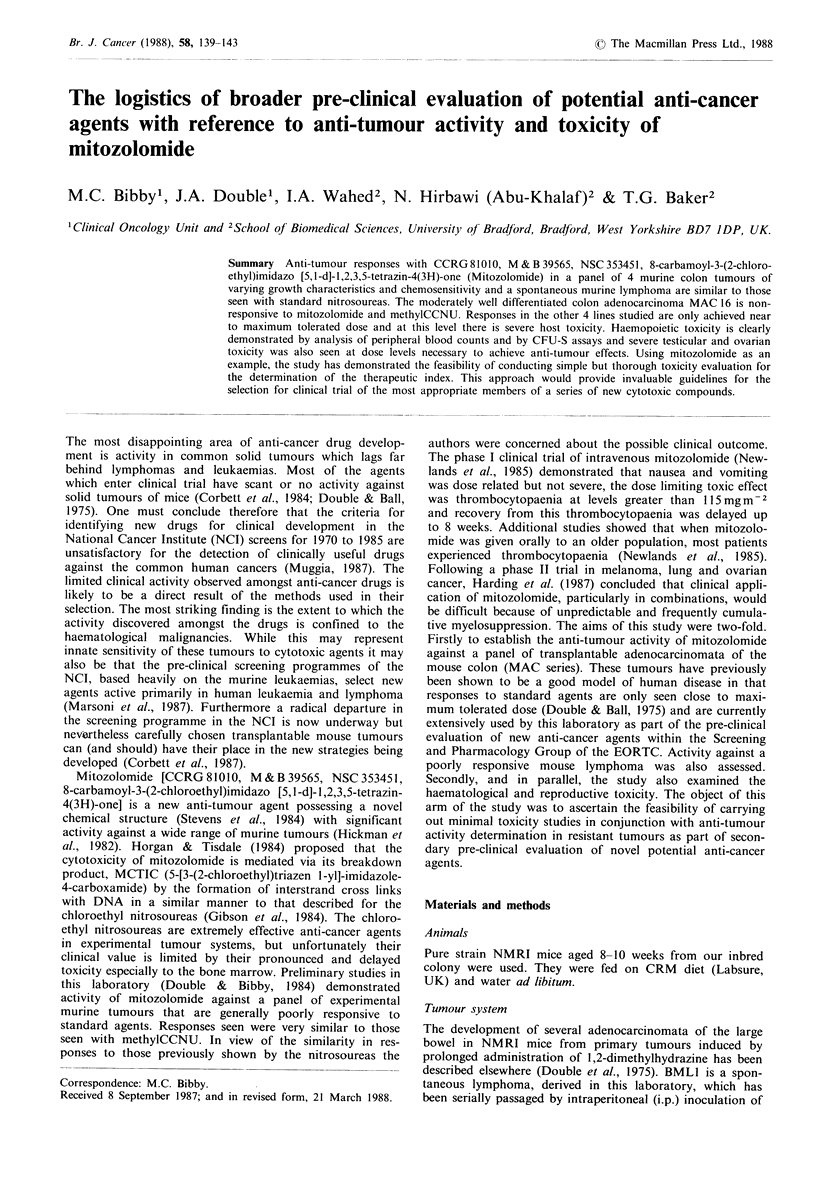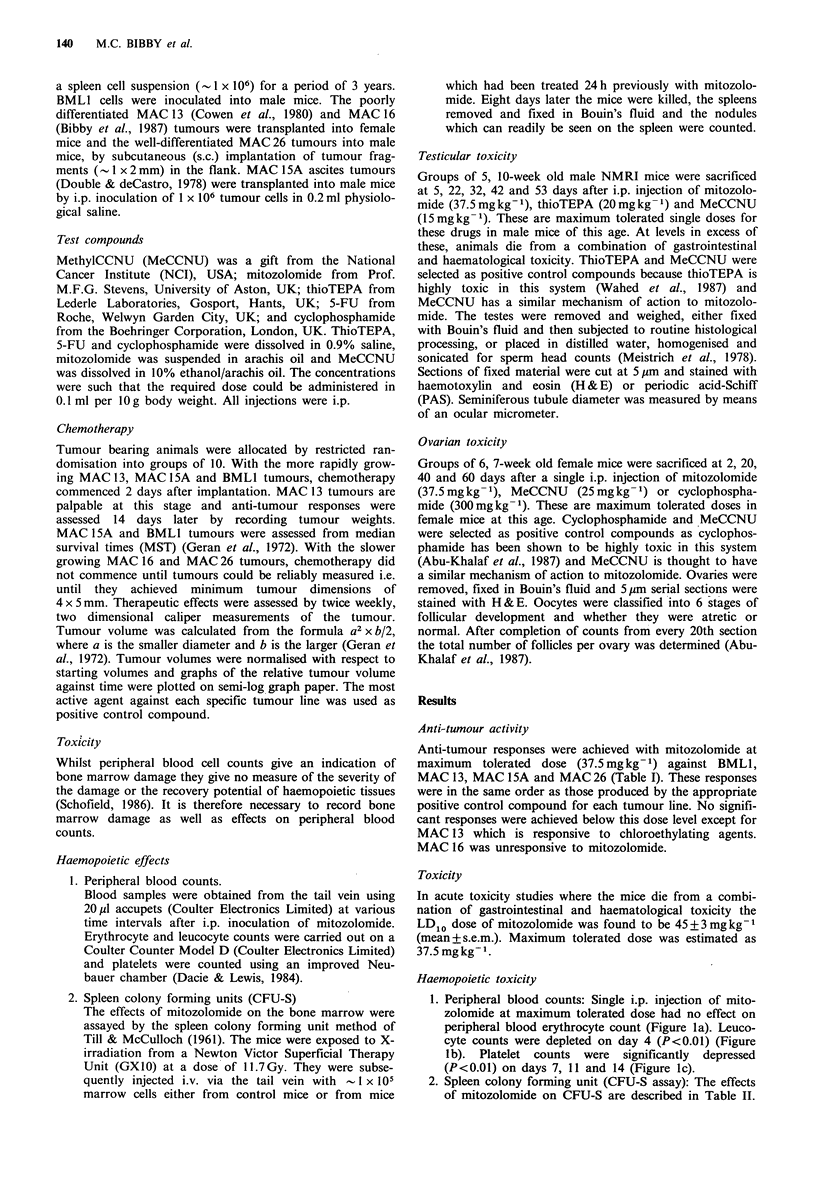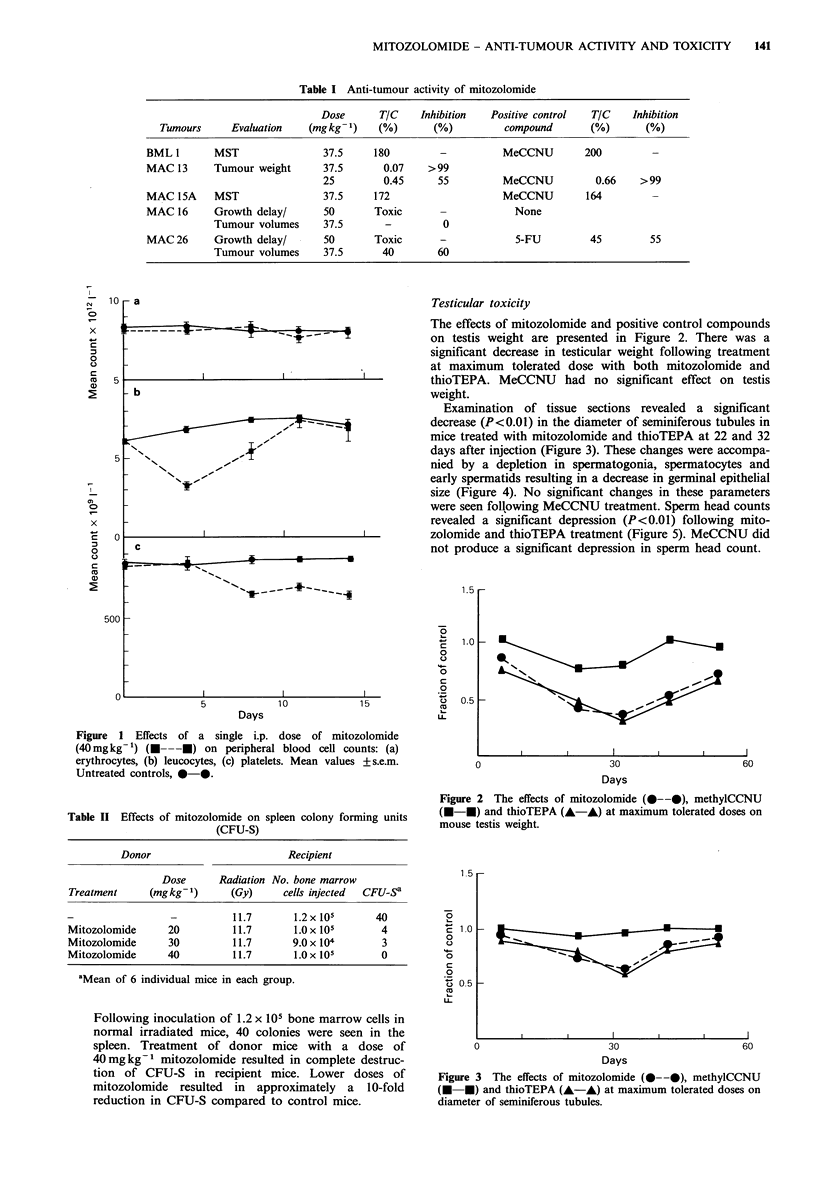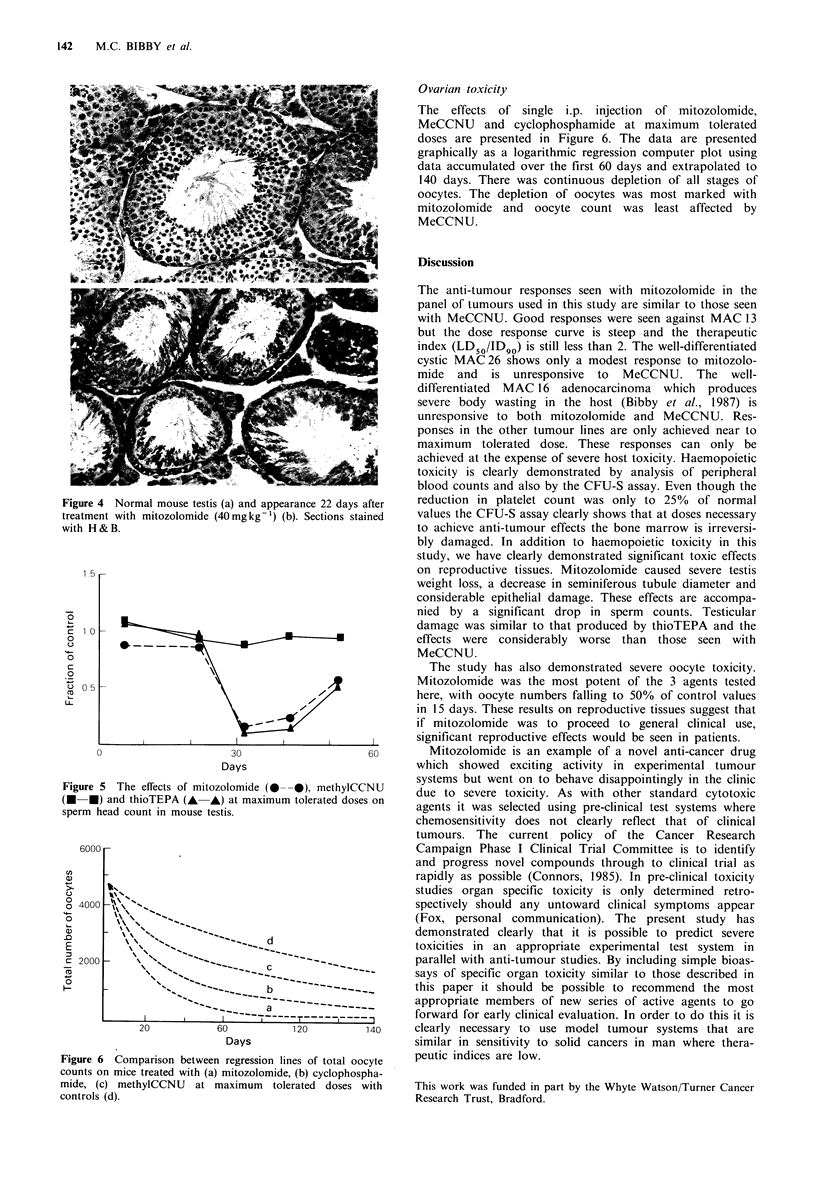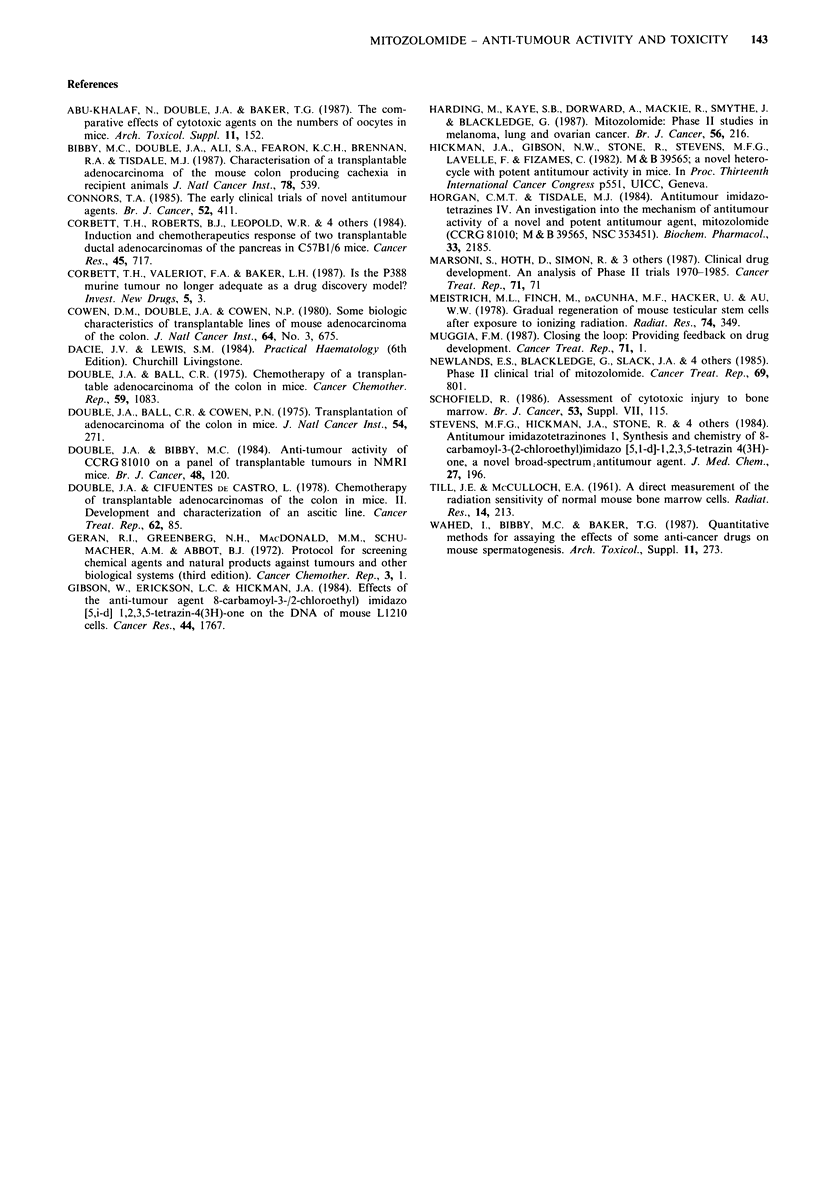# The logistics of broader pre-clinical evaluation of potential anti-cancer agents with reference to anti-tumour activity and toxicity of mitozolomide.

**DOI:** 10.1038/bjc.1988.180

**Published:** 1988-08

**Authors:** M. C. Bibby, J. A. Double, I. A. Wahed, N. Hirbawi, T. G. Baker

**Affiliations:** Clinical Oncology Unit, University of Bradford, West Yorkshire, UK.

## Abstract

**Images:**


Br. J. Cancer (1988), **58**, 139–143 © The Macmillan Press Ltd., 1988

### The logistics of broader pre-clinical evaluation of potential anti-cancer agents with reference to anti-tumour activity and toxicity of mitozolomide

M.C. Bibby[1], J.A. Double[1], I.A. Wahed[2], N. Hirbawi (Abu-Khalaf)[2] & T.G. Baker[2]

[1]*Clinical Oncology Unit and* [2]*School of Biomedical Sciences, University of Bradford, Bradford, West Yorkshire BD7 1DP, UK.*

**Summary** Anti-tumour responses with CCRG 81010, M & B 39565, NSC 353451, 8-carbamoyl-3-(2-chloroethyl)imidazo [5,1-d]-1,2,3,5-tetrazin-4(3H)-one (Mitozolomide) in a panel of 4 murine colon tumours of varying growth characteristics and chemosensitivity and a spontaneous murine lymphoma are similar to those seen with standard nitrosoureas. The moderately well differentiated colon adenocarcinoma MAC 16 is non-responsive to mitozolomide and methylCCNU. Responses in the other 4 lines studied are only achieved near to maximum tolerated dose and at this level there is severe host toxicity. Haemopoietic toxicity is clearly demonstrated by analysis of peripheral blood counts and by CFU-S assays and severe testicular and ovarian toxicity was also seen at dose levels necessary to achieve anti-tumour effects. Using mitozolomide as an example, the study has demonstrated the feasibility of conducting simple but thorough toxicity evaluation for the determination of the therapeutic index. This approach would provide invaluable guidelines for the selection for clinical trial of the most appropriate members of a series of new cytotoxic compounds.

The most disappointing area of anti-cancer drug development is activity in common solid tumours which lags far behind lymphomas and leukaemias. Most of the agents which enter clinical trial have scant or no activity against solid tumours of mice (Corbett *et al.*, 1984; Double & Ball, 1975). One must conclude therefore that the criteria for identifying new drugs for clinical development in the National Cancer Institute (NCI) screens for 1970 to 1985 are unsatisfactory for the detection of clinically useful drugs against the common human cancers (Muggia, 1987). The limited clinical activity observed amongst anti-cancer drugs is likely to be a direct result of the methods used in their selection. The most striking finding is the extent to which the activity discovered amongst the drugs is confined to the haematological malignancies. While this may represent innate sensitivity of these tumours to cytotoxic agents it may also be that the pre-clinical screening programmes of the NCI, based heavily on the murine leukaemias, select new agents active primarily in human leukaemia and lymphoma (Marsoni *et al.*, 1987). Furthermore a radical departure in the screening programme in the NCI is now underway but nevertheless carefully chosen transplantable mouse tumours can (and should) have their place in the new strategies being developed (Corbett *et al.*, 1987).

Mitozolomide [CCRG 81010, M & B 39565, NSC 353451, 8-carbamoyl-3-(2-chloroethyl)imidazo [5,1-d]-1,2,3,5-tetrazin-4(3H)-one] is a new anti-tumour agent possessing a novel chemical structure (Stevens *et al.*, 1984) with significant activity against a wide range of murine tumours (Hickman *et al.*, 1982). Horgan & Tisdale (1984) proposed that the cytotoxicity of mitozolomide is mediated via its breakdown product, MCTIC (5-[3-(2-chloroethyl)triazen 1-yl]-imidazole-4-carboxamide) by the formation of interstrand cross links with DNA in a similar manner to that described for the chloroethyl nitrosoureas (Gibson *et al.*, 1984). The chloroethyl nitrosoureas are extremely effective anti-cancer agents in experimental tumour systems, but unfortunately their clinical value is limited by their pronounced and delayed toxicity especially to the bone marrow. Preliminary studies in this laboratory (Double & Bibby, 1984) demonstrated activity of mitozolomide against a panel of experimental murine tumours that are generally poorly responsive to standard agents. Responses seen were very similar to those seen with methylCCNU. In view of the similarity in responses to those previously shown by the nitrosoureas the authors were concerned about the possible clinical outcome. The phase I clinical trial of intravenous mitozolomide (Newlands *et al.*, 1985) demonstrated that nausea and vomiting was dose related but not severe, the dose limiting toxic effect was thrombocytopaenia at levels greater than $115\,mg\,m^{-2}$ and recovery from this thrombocytopaenia was delayed up to 8 weeks. Additional studies showed that when mitozolomide was given orally to an older population, most patients experienced thrombocytopaenia (Newlands *et al.*, 1985). Following a phase II trial in melanoma, lung and ovarian cancer, Harding *et al.* (1987) concluded that clinical application of mitozolomide, particularly in combinations, would be difficult because of unpredictable and frequently cumulative myelosuppression. The aims of this study were two-fold. Firstly to establish the anti-tumour activity of mitozolomide against a panel of transplantable adenocarcinomata of the mouse colon (MAC series). These tumours have previously been shown to be a good model of human disease in that responses to standard agents are only seen close to maximum tolerated dose (Double & Ball, 1975) and are currently extensively used by this laboratory as part of the pre-clinical evaluation of new anti-cancer agents within the Screening and Pharmacology Group of the EORTC. Activity against a poorly responsive mouse lymphoma was also assessed. Secondly, and in parallel, the study also examined the haematological and reproductive toxicity. The object of this arm of the study was to ascertain the feasibility of carrying out minimal toxicity studies in conjunction with anti-tumour activity determination in resistant tumours as part of secondary pre-clinical evaluation of novel potential anti-cancer agents.

### Materials and methods

### *Animals*

Pure strain NMRI mice aged 8–10 weeks from our inbred colony were used. They were fed on CRM diet (Labsure, UK) and water *ad libitum*.

### *Tumour system*

The development of several adenocarcinomata of the large bowel in NMRI mice from primary tumours induced by prolonged administration of 1,2-dimethylhydrazine has been described elsewhere (Double *et al.*, 1975). BML1 is a spontaneous lymphoma, derived in this laboratory, which has been serially passaged by intraperitoneal (i.p.) inoculation of

Correspondence: M.C. Bibby.
Received 8 September 1987; and in revised form, 21 March 1988.

a spleen cell suspension ($\sim 1 \times 10^6$) for a period of 3 years. BML1 cells were inoculated into male mice. The poorly differentiated MAC 13 (Cowen *et al.*, 1980) and MAC 16 (Bibby *et al.*, 1987) tumours were transplanted into female mice and the well-differentiated MAC 26 tumours into male mice, by subcutaneous (s.c.) implantation of tumour fragments ($\sim 1 \times 2$ mm) in the flank. MAC 15A ascites tumours (Double & deCastro, 1978) were transplanted into male mice by i.p. inoculation of $1 \times 10^6$ tumour cells in 0.2 ml physiological saline.

*Test compounds*

MethylCCNU (MeCCNU) was a gift from the National Cancer Institute (NCI), USA; mitozolomide from Prof. M.F.G. Stevens, University of Aston, UK; thioTEPA from Lederle Laboratories, Gosport, Hants, UK; 5-FU from Roche, Welwyn Garden City, UK; and cyclophosphamide from the Boehringer Corporation, London, UK. ThioTEPA, 5-FU and cyclophosphamide were dissolved in 0.9% saline, mitozolomide was suspended in arachis oil and MeCCNU was dissolved in 10% ethanol/arachis oil. The concentrations were such that the required dose could be administered in 0.1 ml per 10 g body weight. All injections were i.p.

*Chemotherapy*

Tumour bearing animals were allocated by restricted randomisation into groups of 10. With the more rapidly growing MAC 13, MAC 15A and BML1 tumours, chemotherapy commenced 2 days after implantation. MAC 13 tumours are palpable at this stage and anti-tumour responses were assessed 14 days later by recording tumour weights. MAC 15A and BML1 tumours were assessed from median survival times (MST) (Geran *et al.*, 1972). With the slower growing MAC 16 and MAC 26 tumours, chemotherapy did not commence until tumours could be reliably measured i.e. until they achieved minimum tumour dimensions of $4 \times 5$ mm. Therapeutic effects were assessed by twice weekly, two dimensional caliper measurements of the tumour. Tumour volume was calculated from the formula $a^2 \times b/2$, where $a$ is the smaller diameter and $b$ is the larger (Geran *et al.*, 1972). Tumour volumes were normalised with respect to starting volumes and graphs of the relative tumour volume against time were plotted on semi-log graph paper. The most active agent against each specific tumour line was used as positive control compound.

*Toxicity*

Whilst peripheral blood cell counts give an indication of bone marrow damage they give no measure of the severity of the damage or the recovery potential of haemopoietic tissues (Schofield, 1986). It is therefore necessary to record bone marrow damage as well as effects on peripheral blood counts.

*Haemopoietic effects*

1. Peripheral blood counts.
Blood samples were obtained from the tail vein using 20 $\mu$l accupets (Coulter Electronics Limited) at various time intervals after i.p. inoculation of mitozolomide. Erythrocyte and leucocyte counts were carried out on a Coulter Counter Model D (Coulter Electronics Limited) and platelets were counted using an improved Neubauer chamber (Dacie & Lewis, 1984).

2. Spleen colony forming units (CFU-S)
The effects of mitozolomide on the bone marrow were assayed by the spleen colony forming unit method of Till & McCulloch (1961). The mice were exposed to X-irradiation from a Newton Victor Superficial Therapy Unit (GX10) at a dose of 11.7 Gy. They were subsequently injected i.v. via the tail vein with $\sim 1 \times 10^5$ marrow cells either from control mice or from mice which had been treated 24 h previously with mitozolomide. Eight days later the mice were killed, the spleens removed and fixed in Bouin's fluid and the nodules which can readily be seen on the spleen were counted.

*Testicular toxicity*

Groups of 5, 10-week old male NMRI mice were sacrificed at 5, 22, 32, 42 and 53 days after i.p. injection of mitozolomide (37.5 mg kg$^{-1}$), thioTEPA (20 mg kg$^{-1}$) and MeCCNU (15 mg kg$^{-1}$). These are maximum tolerated single doses for these drugs in male mice of this age. At levels in excess of these, animals die from a combination of gastrointestinal and haematological toxicity. ThioTEPA and MeCCNU were selected as positive control compounds because thioTEPA is highly toxic in this system (Wahed *et al.*, 1987) and MeCCNU has a similar mechanism of action to mitozolomide. The testes were removed and weighed, either fixed with Bouin's fluid and then subjected to routine histological processing, or placed in distilled water, homogenised and sonicated for sperm head counts (Meistrich *et al.*, 1978). Sections of fixed material were cut at 5 $\mu$m and stained with haemotoxylin and eosin (H & E) or periodic acid-Schiff (PAS). Seminiferous tubule diameter was measured by means of an ocular micrometer.

*Ovarian toxicity*

Groups of 6, 7-week old female mice were sacrificed at 2, 20, 40 and 60 days after a single i.p. injection of mitozolomide (37.5 mg kg$^{-1}$), MeCCNU (25 mg kg$^{-1}$) or cyclophosphamide (300 mg kg$^{-1}$). These are maximum tolerated doses in female mice at this age. Cyclophosphamide and MeCCNU were selected as positive control compounds as cyclophosphamide has been shown to be highly toxic in this system (Abu-Khalaf *et al.*, 1987) and MeCCNU is thought to have a similar mechanism of action to mitozolomide. Ovaries were removed, fixed in Bouin's fluid and 5 $\mu$m serial sections were stained with H & E. Oocytes were classified into 6 stages of follicular development and whether they were atretic or normal. After completion of counts from every 20th section the total number of follicles per ovary was determined (Abu-Khalaf *et al.*, 1987).

### Results

*Anti-tumour activity*

Anti-tumour responses were achieved with mitozolomide at maximum tolerated dose (37.5 mg kg$^{-1}$) against BML1, MAC 13, MAC 15A and MAC 26 (Table I). These responses were in the same order as those produced by the appropriate positive control compound for each tumour line. No significant responses were achieved below this dose level except for MAC 13 which is responsive to chloroethylating agents. MAC 16 was unresponsive to mitozolomide.

*Toxicity*

In acute toxicity studies where the mice die from a combination of gastrointestinal and haematological toxicity the $LD_{10}$ dose of mitozolomide was found to be $45 \pm 3$ mg kg$^{-1}$ (mean $\pm$ s.e.m.). Maximum tolerated dose was estimated as 37.5 mg kg$^{-1}$.

*Haemopoietic toxicity*

1. Peripheral blood counts: Single i.p. injection of mitozolomide at maximum tolerated dose had no effect on peripheral blood erythrocyte count (Figure 1a). Leucocyte counts were depleted on day 4 ($P<0.01$) (Figure 1b). Platelet counts were significantly depressed ($P<0.01$) on days 7, 11 and 14 (Figure 1c).
2. Spleen colony forming unit (CFU-S assay): The effects of mitozolomide on CFU-S are described in Table II.

**Table I** Anti-tumour activity of mitozolomide

| *Tumours* | *Evaluation* | *Dose* *(mg kg$^{-1}$)* | *T/C* *(%)* | *Inhibition* *(%)* | *Positive control* *compound* | *T/C* *(%)* | *Inhibition* *(%)* |
|---|---|---|---|---|---|---|---|
| BML 1 | MST | 37.5 | 180 | – | MeCCNU | 200 | – |
| MAC 13 | Tumour weight | 37.5 | 0.07 | >99 | | | |
| | | 25 | 0.45 | 55 | MeCCNU | 0.66 | >99 |
| MAC 15A | MST | 37.5 | 172 | | MeCCNU | 164 | – |
| MAC 16 | Growth delay/ | 50 | Toxic | – | None | | |
| | Tumour volumes | 37.5 | – | 0 | | | |
| MAC 26 | Growth delay/ | 50 | Toxic | – | 5-FU | 45 | 55 |
| | Tumour volumes | 37.5 | 40 | 60 | | | |

**Figure 1** Effects of a single i.p. dose of mitozolomide (40 mg kg$^{-1}$) (■---■) on peripheral blood cell counts: (a) erythrocytes, (b) leucocytes, (c) platelets. Mean values ± s.e.m. Untreated controls, ●—●.

**Table II** Effects of mitozolomide on spleen colony forming units (CFU-S)

| *Donor* | | *Recipient* | | |
|---|---|---|---|---|
| *Treatment* | *Dose* *(mg kg$^{-1}$)* | *Radiation* *(Gy)* | *No. bone marrow* *cells injected* | *CFU-S*[a] |
| – | – | 11.7 | $1.2 \times 10^5$ | 40 |
| Mitozolomide | 20 | 11.7 | $1.0 \times 10^5$ | 4 |
| Mitozolomide | 30 | 11.7 | $9.0 \times 10^4$ | 3 |
| Mitozolomide | 40 | 11.7 | $1.0 \times 10^5$ | 0 |

[a]Mean of 6 individual mice in each group.

Following inoculation of $1.2 \times 10^5$ bone marrow cells in normal irradiated mice, 40 colonies were seen in the spleen. Treatment of donor mice with a dose of 40 mg kg$^{-1}$ mitozolomide resulted in complete destruction of CFU-S in recipient mice. Lower doses of mitozolomide resulted in approximately a 10-fold reduction in CFU-S compared to control mice.

*Testicular toxicity*

The effects of mitozolomide and positive control compounds on testis weight are presented in Figure 2. There was a significant decrease in testicular weight following treatment at maximum tolerated dose with both mitozolomide and thioTEPA. MeCCNU had no significant effect on testis weight.

Examination of tissue sections revealed a significant decrease ($P < 0.01$) in the diameter of seminiferous tubules in mice treated with mitozolomide and thioTEPA at 22 and 32 days after injection (Figure 3). These changes were accompanied by a depletion in spermatogonia, spermatocytes and early spermatids resulting in a decrease in germinal epithelial size (Figure 4). No significant changes in these parameters were seen following MeCCNU treatment. Sperm head counts revealed a significant depression ($P < 0.01$) following mitozolomide and thioTEPA treatment (Figure 5). MeCCNU did not produce a significant depression in sperm head count.

**Figure 2** The effects of mitozolomide (●--●), methylCCNU (■—■) and thioTEPA (▲—▲) at maximum tolerated doses on mouse testis weight.

**Figure 3** The effects of mitozolomide (●--●), methylCCNU (■—■) and thioTEPA (▲—▲) at maximum tolerated doses on diameter of seminiferous tubules.

**Figure 4** Normal mouse testis (a) and appearance 22 days after treatment with mitozolomide (40 mg kg$^{-1}$) (b). Sections stained with H & B.

**Figure 5** The effects of mitozolomide (●--●), methylCCNU (■—■) and thioTEPA (▲—▲) at maximum tolerated doses on sperm head count in mouse testis.

**Figure 6** Comparison between regression lines of total oocyte counts on mice treated with (a) mitozolomide, (b) cyclophosphamide, (c) methylCCNU at maximum tolerated doses with controls (d).

*Ovarian toxicity*

The effects of single i.p. injection of mitozolomide, MeCCNU and cyclophosphamide at maximum tolerated doses are presented in Figure 6. The data are presented graphically as a logarithmic regression computer plot using data accumulated over the first 60 days and extrapolated to 140 days. There was continuous depletion of all stages of oocytes. The depletion of oocytes was most marked with mitozolomide and oocyte count was least affected by MeCCNU.

### Discussion

The anti-tumour responses seen with mitozolomide in the panel of tumours used in this study are similar to those seen with MeCCNU. Good responses were seen against MAC 13 but the dose response curve is steep and the therapeutic index ($LD_{50}/ID_{90}$) is still less than 2. The well-differentiated cystic MAC 26 shows only a modest response to mitozolomide and is unresponsive to MeCCNU. The well-differentiated MAC 16 adenocarcinoma which produces severe body wasting in the host (Bibby *et al.*, 1987) is unresponsive to both mitozolomide and MeCCNU. Responses in the other tumour lines are only achieved near to maximum tolerated dose. These responses can only be achieved at the expense of severe host toxicity. Haemopoietic toxicity is clearly demonstrated by analysis of peripheral blood counts and also by the CFU-S assay. Even though the reduction in platelet count was only to 25% of normal values the CFU-S assay clearly shows that at doses necessary to achieve anti-tumour effects the bone marrow is irreversibly damaged. In addition to haemopoietic toxicity in this study, we have clearly demonstrated significant toxic effects on reproductive tissues. Mitozolomide caused severe testis weight loss, a decrease in seminiferous tubule diameter and considerable epithelial damage. These effects are accompanied by a significant drop in sperm counts. Testicular damage was similar to that produced by thioTEPA and the effects were considerably worse than those seen with MeCCNU.

The study has also demonstrated severe oocyte toxicity. Mitozolomide was the most potent of the 3 agents tested here, with oocyte numbers falling to 50% of control values in 15 days. These results on reproductive tissues suggest that if mitozolomide was to proceed to general clinical use, significant reproductive effects would be seen in patients.

Mitozolomide is an example of a novel anti-cancer drug which showed exciting activity in experimental tumour systems but went on to behave disappointingly in the clinic due to severe toxicity. As with other standard cytotoxic agents it was selected using pre-clinical test systems where chemosensitivity does not clearly reflect that of clinical tumours. The current policy of the Cancer Research Campaign Phase I Clinical Trial Committee is to identify and progress novel compounds through to clinical trial as rapidly as possible (Connors, 1985). In pre-clinical toxicity studies organ specific toxicity is only determined retrospectively should any untoward clinical symptoms appear (Fox, personal communication). The present study has demonstrated clearly that it is possible to predict severe toxicities in an appropriate experimental test system in parallel with anti-tumour studies. By including simple bioassays of specific organ toxicity similar to those described in this paper it should be possible to recommend the most appropriate members of new series of active agents to go forward for early clinical evaluation. In order to do this it is clearly necessary to use model tumour systems that are similar in sensitivity to solid cancers in man where therapeutic indices are low.

This work was funded in part by the Whyte Watson/Turner Cancer Research Trust, Bradford.

### References

ABU-KHALAF, N., DOUBLE, J.A. & BAKER, T.G. (1987). The comparative effects of cytotoxic agents on the numbers of oocytes in mice. *Arch. Toxicol. Suppl.* **11**, 152.

BIBBY, M.C., DOUBLE, J.A., ALI, S.A., FEARON, K.C.H., BRENNAN, R.A. & TISDALE, M.J. (1987). Characterisation of a transplantable adenocarcinoma of the mouse colon producing cachexia in recipient animals *J. Natl Cancer Inst.*, **78**, 539.

CONNORS, T.A. (1985). The early clinical trials of novel antitumour agents. *Br. J. Cancer*, **52**, 411.

CORBETT, T.H., ROBERTS, B.J., LEOPOLD, W.R. & 4 others (1984). Induction and chemotherapeutics response of two transplantable ductal adenocarcinomas of the pancreas in C57B1/6 mice. *Cancer Res.*, **45**, 717.

CORBETT, T.H., VALERIOT, F.A. & BAKER, L.H. (1987). Is the P388 murine tumour no longer adequate as a drug discovery model? *Invest. New Drugs*, **5**, 3.

COWEN, D.M., DOUBLE, J.A. & COWEN, N.P. (1980). Some biologic characteristics of transplantable lines of mouse adenocarcinoma of the colon. *J. Natl Cancer Inst.*, **64**, No. 3, 675.

DACIE, J.V. & LEWIS, S.M. (1984). *Practical Haematology* (6th Edition). Churchill Livingstone.

DOUBLE, J.A. & BALL, C.R. (1975). Chemotherapy of a transplantable adenocarcinoma of the colon in mice. *Cancer Chemother. Rep.*, **59**, 1083.

DOUBLE, J.A., BALL, C.R. & COWEN, P.N. (1975). Transplantation of adenocarcinoma of the colon in mice. *J. Natl Cancer Inst.*, **54**, 271.

DOUBLE, J.A. & BIBBY, M.C. (1984). Anti-tumour activity of CCRG 81010 on a panel of transplantable tumours in NMRI mice. *Br. J. Cancer*, **48**, 120.

DOUBLE, J.A. & CIFUENTES DE CASTRO, L. (1978). Chemotherapy of transplantable adenocarcinomas of the colon in mice. II. Development and characterization of an ascitic line. *Cancer Treat. Rep.*, **62**, 85.

GERAN, R.I., GREENBERG, N.H., MACDONALD, M.M., SCHUMACHER, A.M. & ABBOT, B.J. (1972). Protocol for screening chemical agents and natural products against tumours and other biological systems (third edition). *Cancer Chemother. Rep.*, **3**, 1.

GIBSON, W., ERICKSON, L.C. & HICKMAN, J.A. (1984). Effects of the anti-tumour agent 8-carbamoyl-3-/2-chloroethyl) imidazo [5,i-d] 1,2,3,5-tetrazin-4(3H)-one on the DNA of mouse L1210 cells. *Cancer Res.*, **44**, 1767.

HARDING, M., KAYE, S.B., DORWARD, A., MACKIE, R., SMYTHE, J. & BLACKLEDGE, G. (1987). Mitozolomide: Phase II studies in melanoma, lung and ovarian cancer. *Br. J. Cancer*, **56**, 216.

HICKMAN, J.A., GIBSON, N.W., STONE, R., STEVENS, M.F.G., LAVELLE, F. & FIZAMES, C. (1982). M & B 39565; a novel heterocycle with potent antitumour activity in mice. In *Proc. Thirteenth International Cancer Congress* p551, UICC, Geneva.

HORGAN, C.M.T. & TISDALE, M.J. (1984). Antitumour imidazotetrazines IV. An investigation into the mechanism of antitumour activity of a novel and potent antitumour agent, mitozolomide (CCRG 81010; M & B 39565, NSC 353451). *Biochem. Pharmacol.*, **33**, 2185.

MARSONI, S., HOTH, D., SIMON, R. & 3 others (1987). Clinical drug development. An analysis of Phase II trials 1970–1985. *Cancer Treat. Rep.*, **71**, 71

MEISTRICH, M.L., FINCH, M., DACUNHA, M.F., HACKER, U. & AU, W.W. (1978). Gradual regeneration of mouse testicular stem cells after exposure to ionizing radiation. *Radiat. Res.*, **74**, 349.

MUGGIA, F.M. (1987). Closing the loop: Providing feedback on drug development. *Cancer Treat. Rep.*, **71**, 1.

NEWLANDS, E.S., BLACKLEDGE, G., SLACK, J.A. & 4 others (1985). Phase II clinical trial of mitozolomide. *Cancer Treat. Rep.*, **69**, 801.

SCHOFIELD, R. (1986). Assessment of cytotoxic injury to bone marrow. *Br. J. Cancer*, **53**, Suppl. VII, 115.

STEVENS, M.F.G., HICKMAN, J.A., STONE, R. & 4 others (1984). Antitumour imidazotetrazinones 1, Synthesis and chemistry of 8-carbamoyl-3-(2-chloroethyl)imidazo [5,1-d]-1,2,3,5-tetrazin 4(3H)-one, a novel broad-spectrum antitumour agent. *J. Med. Chem.*, **27**, 196.

TILL, J.E. & MCCULLOCH, E.A. (1961). A direct measurement of the radiation sensitivity of normal mouse bone marrow cells. *Radiat. Res.*, **14**, 213.

WAHED, I., BIBBY, M.C. & BAKER, T.G. (1987). Quantitative methods for assaying the effects of some anti-cancer drugs on mouse spermatogenesis. *Arch. Toxicol.*, Suppl. **11**, 273.